# Nebulized Tranexamic Acid for Pediatric Post-tonsillectomy Hemorrhage: A Report of Two Cases

**DOI:** 10.5811/cpcem.2021.2.50799

**Published:** 2021-03-12

**Authors:** Cecilia Monteilh, Lydia Rabon, Ilana Mayer-Hirshfeld, Jon McGreevy

**Affiliations:** * Phoenix Children’s Hospital, Department of Emergency Medicine, Phoenix, Arizona; † Valleywise Health Medical Center, Department of Emergency Medicine, Phoenix, Arizona

**Keywords:** post-tonsillectomy hemorrhage, nebulized tranexamic acid, tranexamic acid

## Abstract

**Introduction:**

Tranexamic acid (TXA) use in pediatrics to control hemorrhage has gained interest in recent years, but there is limited literature on nebulized TXA especially regarding dosing and adverse effects. Tranexamic acid has anti-fibrinolytic properties via competitive inhibition of plasminogen activation making it a logical approach to promote hemostasis in cases of post-tonsillectomy hemorrhage.

**Case Report:**

We describe two cases of post-tonsillectomy hemorrhage managed with nebulized TXA. In both cases, bleeding was stopped after TXA administration.

**Conclusion:**

To our knowledge, this is the first case report to describe the use of nebulized TXA without an adjunct pharmacotherapy. Our two cases add additional reportable data on the safety of nebulized TXA and possible effectiveness on post-tonsillectomy hemorrhage.

## INTRODUCTION

Tonsillectomy is one of the most common surgical procedures in pediatrics. Approximately 1–10% of patients who have tonsillectomies will have their course complicated by post-tonsillectomy hemorrhage.^1^–^3^ Post-tonsillectomy hemorrhages are defined as primary or secondary based on their timing. Primary hemorrhage occurs within the first 24 hours postoperatively.^1^,^2^ Secondary hemorrhage occurs after the first 24-hour period, most commonly between days five and ten, usually from sloughing of the eschar, trauma due to food ingestion, or infection of the tonsil bed, among other causes.^1^,^2^ Most of these hemorrhages are self-limited, but some are serious and can lead to death from hemorrhagic shock or airway obstruction.^4^

If on presentation to the emergency department (ED) the patient is actively bleeding, the treatment modalities consist of ice water gargles, topical or racemic epinephrine, and applying direct pressure.^1^ These treatments are not always effective, which then necessitates a return to the operating room (OR). Recent research supports the use of tranexamic acid (TXA) for treating a variety of bleeding issues following surgical procedures, but there are only two case reports of its use in post-tonsillectomy bleeding. One of these cases was in an adult patient and the other in a pediatric patient.^1^,^5^ Our goal in reporting this case was to add evidence supporting the use of nebulized TXA in post-tonsillectomy hemorrhages.

## CASE REPORT

### Case 1

A three-year-old male with a history of obstructive sleep apnea presented to the pediatric emergency department (PED) on postoperative day (POD) three with a chief complaint of hematemesis. The morning of presentation, the patient awoke fussy and subsequently began to vomit. His mother witnessed a clot in the emesis with frank hematemesis with continued bleeding coming from the oropharynx, and she transported him via personal vehicle to the hospital. Upon arrival, the patient was afebrile at 37.5ºC, with a heart rate of 137 beats per minute, respiratory rate of 28 breaths per minute, oxygen saturation of 98% on room air, blood pressure of 111/85 millimeters mercury (mm Hg), and weight of 12 kilograms (kg). On exam, the patient appeared pale and tachycardic with active retching, and with blood pooled in the posterior oropharynx that obscured visualization of the tonsillar beds.

A complete blood count (CBC), coagulation profile, and type and cross were obtained, and ondansetron was administered intravenously (IV). A cold-water rinse was attempted; however, the patient did not tolerate this intervention. Nebulized TXA was administered by using 250 milligrams (mg) (50mg/per milliliter) IV solution via direct nebulization at a flow rate of eight liters over four minutes without additive of normal saline solution. After administration of TXA, the patient had no obvious bleeding from the posterior pharynx and his retching improved. He was then admitted to the general pediatric inpatient service for further monitoring with pediatric otolaryngology (ENT) consultation. The CBC and coagulation studies were within the normal range on admission with hemoglobin 12.0 grams per deciliter (gm/dL) (normal range 11.5–14.5 gm/dL), prothrombin time (PT) 13.8 seconds (sec) (11.7–14.7 sec), and partial thromboplastin time (PTT) 33.3 sec (25–35 sec).

These lab tests were not repeated during his hospitalization. After an initial refusal of oral intake requiring maintenance IV fluids the patient was able to be transitioned to a soft diet, which he tolerated, and he was discharged on hospital day three. He had no further bleeding, and no adverse effects from nebulized TXA were documented. The patient’s initial surgery was performed at an outside facility, and no follow-up visits were done at our institution for review.

### Case 2

A two-year-old male presented to the PED on POD two with a chief complaint of hematemesis and concern for active post-tonsillectomy hemorrhage. The patient was accompanied by his mother. She stated that the patient had been taking medication approximately one hour before arrival, began to gag, and then had an episode of hematemesis. The mother immediately transported the patient via personal vehicle to the PED with two more episodes of hematemesis en route. On arrival, the patient was afebrile at 36.7ºC, with a heart rate of 150 beats per minute, oxygen saturation of 99% on room air, blood pressure of 90/60 mm Hg, and weight of 14.2 kg. On exam, he appeared pale and in distress in triage, and so was taken to a resuscitation room where an IV was established, and a normal saline bolus (20 cubic centimeters/kg) was initiated.

Active bleeding of the tonsillar bed was appreciated with blood pooling in the posterior oropharynx and there was an inability to appreciate laterality of source, for which ENT was immediately called. During this time, nebulized TXA was administered using 250 mg (50mg/mL) IV solution via direct nebulization without additive of normal saline solution. The bleeding stopped within 10 minutes of nebulized TXA administration. Initial labs resulted as follows: hemoglobin 10.7 gm/dL; PT 15.6 sec; PTT 26.2 sec. The patient was then transported to the OR by ENT for definitive management.

**CPC-EM Capsule**

What do we already know about this clinical entity?

*Recent research supports the use of tranexamic acid (TXA) for bleeding following surgical procedures*.

What makes this presentation of disease reportable?

*Two pediatric patients presented to our emergency department with post-tonsillectomy hemorrhage and were successfully managed with nebulized TXA without adjunct pharmacotherapy*.

What is the major learning point?

*Nebulized TXA may be a viable single-therapy option in managing pediatric post-tonsillectomy hemorrhage*.

How might this improve emergency medicine practice?

*Initiation of nebulized TXA for pediatric post-tonsillectomy hemorrhage may temporize bleeding while awaiting definitive management by otolaryngology*.

The following was noted in the operative report documentation by the surgeon: 1) normal palate; 2) normal postoperative changes of the adenoids without active bleeding; and 3) a clot on the left tonsillar pillar, which was removed. No active bleeding was noted otherwise, and the bilateral fossa was cauterized. The patient was then admitted to the general pediatric inpatient service for further monitoring overnight. A hemoglobin check performed the following morning was 8.4 gm/dL. The patient tolerated a soft diet the next day and was discharged home with return precautions and ENT follow-up. There were no documented adverse effects during hospitalization, and no ENT follow-up documentation was available for our review in the health record despite initial surgery at our facility.

## DISCUSSION

Post-tonsillectomy hemorrhage represents a gray area not only in definition but in management. There are several treatment options with variable success, many of which are complicated in the pediatric patient. This has led to institutional preference and wide variability in management algorithms. Even with a definitive treatment option available and operative management, there is a need for temporizing or bridging measures. As a result of this, TXA has gained attention. Tranexamic acid is an antifibrinolytic that competitively inhibits plasminogen activation, thus promoting hemostasis and decreasing bleeding by forming a reversible complex that displaces plasminogen from fibrin, resulting in inhibition of fibrinolysis. It also works by inhibiting the proteolytic activity of plasmin.^1^,^6^–^9^ Its use in pediatrics has been seen with scoliosis repair, congenital heart repair, and craniosynostosis repair, but has also been increasingly reported in pediatric trauma, such as in the Clinical Randomization of an Antifibrinolytic in Significant Hemorrhage (CRASH-2) trial, diffuse alveolar hemorrhage (DAH), and more recently with post-tonsillectomy hemorrhage.^1^–^11^ As a result of broadening use with limited patient numbers actual dosing parameters are not well established, nor is the safety profile when used for these indications.

The use of TXA as a preventative medication for post-tonsillectomy hemorrhage has been studied with little reported benefit but did show an overall reduction in mean blood loss and mean duration of bleeding intraoperatively.^8^ Systemic administration of TXA has been shown to have minor side effects ranging from nausea and vomiting to more severe side effects such as seizures and renal toxicity in those patients with already decreased function.^7^–^9^ Most of these side effects have only been described in adult patients, and seizures seem to be related to high-dose administration, especially in cardiac surgery.^9^ Maeda et al found that prolonging drug delivery time during the early postoperative period may lead to a reduction in seizure events.^7^ Other studies have found that administration of TXA during tonsillectomy has side effects such as nausea, headache, vomiting, and dizziness.^12^ It appears that these side effects may be dose related, but due to lack of standardized dosing for its various uses this is difficult to extrapolate.^7^

Poppe and Grimaldo described a 22-year-old male who presented to the ED with an active post-tonsillectomy hemorrhage. He became hypotensive and received emergent IV fluids, massive transfusion protocol, and nebulized TXA. After completion of the nebulized TXA, the patient’s bleeding was controlled.^5^ This was the first case in the emergency medicine literature that described the use of nebulized TXA in an adult to achieve hemostasis in post-tonsillectomy hemorrhage. Swartz et al administered nebulized TXA to achieve hemostasis in a pediatric patient with associated bleeding cessation prior to definitive operative management as well.^1^ However, these are the only two case reports describing the use of nebulized TXA to control post-tonsillectomy bleeding.

In the case report by Schwarz et al, dosing was extrapolated from the literature on DAH at 250 mg of nebulized TXA to be used for post-tonsillectomy hemorrhage for children less than 25 kg and 500 mg for those greater than 25 kg after a failed attempt to stop the hemorrhage with nebulized racemic epinephrine.^1^,^11^ Poppe and Grimaldo used nebulized TXA (1000 mg/10 mL) 5 mL and normal saline 5 mL in an adult patient, which allowed for the mitigation of a massive post-tonsillectomy hemorrhage until ENT could arrive at bedside to perform a coagulation cautery.^5^ This dosing was also based on the aforementioned DAH dosing.^11^ In the two previous studies describing nebulized TXA, neither showed any adverse effects locally or systemically.^1^,^2^,^5^ This was our experience as well and supplements the safety data in addition to providing a specific effective dose.

Our two patients did not receive racemic epinephrine prior to the administration of nebulized TXA. This differs from Schwarz et al, where they used racemic epinephrine prior to their TXA trial, which may have introduced a complementary mechanism to the cessation of bleeding in their case.^1^ Our cases demonstrate that nebulized TXA alone may allow for the cessation of post-tonsillectomy hemorrhages without adjunct pharmacotherapy.

## CONCLUSION

To our knowledge, this is the first pediatric case report to describe the use of nebulized TXA without any adjunct pharmacotherapy. Our two cases add additional data on the safety of nebulized TXA and possible effectiveness for the treatment of post-tonsillectomy hemorrhage. A multicenter randomized control trial would be the ideal manner to delineate effectiveness and safety profile, but given the rarity of cases it would be difficult to accomplish especially without preliminary experience to show it as a reasonable treatment modality. However, based on existing data nebulized TXA appears to be a safe and potentially effective option in acute post-tonsillectomy bleeding.
